# Photothermal Therapy as Adjuvant to Surgery in an Orthotopic Mouse Model of Human Fibrosarcoma

**DOI:** 10.3390/cancers13225820

**Published:** 2021-11-20

**Authors:** Marina Simón, Jesper Tranekjær Jørgensen, Fredrik Melander, Thomas Lars Andresen, Anders Christensen, Andreas Kjaer

**Affiliations:** 1 Department of Clinical Physiology, Nuclear Medicine and PET & Cluster for Molecular Imaging, Copenhagen University Hospital—Rigshospitalet & Department of Biomedical Sciences, University of Copenhagen, 2100 Copenhagen, Denmark; marina.simon@sund.ku.dk (M.S.); jespertj@sund.ku.dk (J.T.J.); anders.christensen.03@regionh.dk (A.C.); 2 Department of Health Technology, Section for Biotherapeutic Engineering and Drug Targeting, Technical University of Denmark, 2800 Kongens Lyngby, Denmark; lpfm@dtu.dk (F.M.); tlan@dtu.dk (T.L.A.); 3 Department of Otolaryngology, Head & Neck Surgery and Audiology, Rigshospitalet, 2100 Copenhagen, Denmark

**Keywords:** photothermal therapy (PTT), surgery, fibrosarcoma, magnetic resonance imaging (MRI)

## Abstract

**Simple Summary:**

Combining tumor surgery with other types of treatment can be useful when dealing with aggressive tumors or tumors in difficult locations. Photothermal therapy (PTT) is a technique based on the use of light-absorbing nanoparticles that accumulate in the tumor. When tumors are irradiated with a laser, these nanoparticles transform the laser light into heat, causing very localized tumor death and sparing healthy neighboring tissues. In this study, we evaluated a treatment strategy consisting of surgery followed by PTT in a highly aggressive mouse model of fibrosarcoma. Using magnetic resonance imaging, we observed a slowdown in tumor growth accompanied by improved survival in mice that underwent PTT and surgery compared to animals that only had surgery. This shows the potential of combining PTT with surgery, an approach that can potentially be valuable to multiple types of cancer.

**Abstract:**

Surgery is still the first-line treatment for multiple solid cancers. However, recurrence is a common issue, especially when dealing with aggressive tumors or tumors that are difficult to completely remove due to their location. Getting clear surgical margins can be challenging, but treatment strategies combining surgery with other anti-cancer therapies can potentially improve the outcome. Photothermal therapy (PTT) is a technique that relies on photoabsorbing agents, such as gold nanoparticles, to transform light into local hyperthermia. This technique can be used to ablate tumor tissue where the photoabsorbing agent accumulates, sparing healthy surrounding tissue. In this study, we examined the potential of gold nanoparticle-based PTT as an adjuvant treatment to surgery in a mouse model of human fibrosarcoma. For this we performed subtotal tumor resection to mimic a clinical situation where total tumor removal is not achieved, and subsequent PTT was applied on the surgical field. Our results showed that animals undergoing adjuvant PTT after surgery presented sustained delayed tumor growth and improved survival when compared to animals that only underwent surgery. We believe that these findings show the potential of PTT as an adjuvant method to traditional tumor surgery and could pave way to more personalized treatment options.

## 1. Introduction

Cancer treatment has relied heavily on surgery for many years, and notwithstanding the introduction of many novel therapeutic modalities in oncology, resection is still central to the removal of solid tumors. However, incomplete surgical removal resulting in recurrence remains a major challenge. Treatment regimens combining surgery with other treatment modalities are therefore a persistent and important focus of research, with the aim of improving clinical outcome while minimizing damage to healthy adjacent tissues [1,2,3,4,5].

Photothermal cancer therapy (PTT) is a clinically applied treatment concept based on the use of near-infrared (NIR) light-absorbing nanoparticles, such as gold nanoshells (NS), which accumulate in the tumor and transform light into heat when irradiated, causing irreversible tumor damage [6,7,8,9]. PTT has been studied as a standalone treatment on multiple mouse tumor models. Although PTT has been shown to have a significant treatment effect, many factors are involved in its success (e.g., tumor size, laser power, nanoparticle accumulation, etc.), and it is often insufficient to achieve complete tumor removal [10,11,12]. As a result, studies applying PTT in combination with therapies such as immunotherapy and chemotherapy have recently yielded positive results. Additionally, the use of PTT as an adjuvant treatment to surgery has proven promising in mouse models of breast cancer [13,14,15], showcasing the potential of PTT as a tool to achieve clearance of residual disease when complete surgical tumor resection is not possible due to, for example, the location of the tumor. This is particularly challenging with highly infiltrative tumors.

Fibrosarcoma is a rare and highly malignant subtype of soft tissue sarcoma with low sensitivity to chemo- and radiotherapy [16,17]. Fibrosarcoma mainly occurs in collagen-rich connective tissues from the lower extremities and can also be located in deep soft tissue, usually causing painless swelling, which in turn leads to the tumor being undetected for a long period of time [18]. The diagnosis of fibrosarcoma relies mainly on pathology, but imaging techniques such as computed tomography (CT) and magnetic resonance imaging (MRI) have also proven useful, with MRI being the most valuable imaging method for diagnosis [19]. Due to the ability of the tumor to grow and spread rapidly, complete tumor resection has proven difficult, and recurrence is common [17]. It has previously been shown that when the HT1080 human fibrosarcoma cell line is implanted in the quadriceps femoris of mice, the generated tumor tissue can invade its surroundings, including bone and muscle tissue, which mimics the tumor progression seen in patients. Thus, this model can be used as a valuable tool for preclinical development and evaluation of new strategies for surgical resection before translation to the clinic [20].

In this study, the aim was to assess the feasibility of applying PTT as an adjuvant to surgery to improve the outcome in a highly invasive human fibrosarcoma model in mice. For this, mice bearing subcutaneous or orthotopic HT1080 tumors were injected with gold NS and underwent subtotal tumor resection to mimic a clinical setting where complete tumor removal is not achieved [1,21]. Following this, the surgical field was exposed to NIR light to perform PTT and the effect of the treatment was monitored over time by measuring changes in tumor volume. 

## 2. Materials and Methods

### 2.1. Gold Nanoshells

800 nm Resonant Biopure™ Gold NS (lot number JLF0015, NanoComposix Europe, Prague, Czech Republic) were used. They are composed of a silica core surrounded by a thin gold shell and functionalized with a 5 KDa poly(ethylene glycol). The diameter of the NS is 150 ± 9 nm, and the diameter of the core is 119 ± 5 nm, as reported by the supplier.

### 2.2. Cell Line and Animal Model

The animal experiments were approved by the Danish Animal Experimentation Council (2016-15-0201-00920) and undertaken in compliance with the directive 2010/63/EU of the EU legislation on the protection of animals used for scientific purposes.

HT1080 human fibrosarcoma cells (ATCC) were cultured in Dulbecco’s modified Eagle’s medium supplemented with 10% fetal bovine serum and 1% penicillin-streptomycin (Thermo Fisher Scientific, Waltham, MA, USA) and kept at 37 °C in 5% CO_2_. For the orthotopic model, 4 × 10^5^ cells in 20 μL PBS were injected into muscle tissue located in the left leg of female NMRI nude mice (Janvier, Genest St. Isle, France). For the subcutaneous model, 2–3 × 10^6^ cells in 100 μL PBS were injected subcutaneously into the left flank. Mice were kept under anesthesia throughout the inoculation process by breathing 4% sevoflurane.

### 2.3. Surgical Resection Followed by Photothermal Therapy

Mice bearing subcutaneous or orthotopic tumors were divided into three groups, matching tumor sizes; surgery + NS (mice injected intravenously with NS and undergoing PTT and surgery), surgery + saline (mice injected intravenously with saline and undergoing PTT and surgery) and surgery (mice undergoing surgery but not PTT). One day before treatment (day −1), mice were injected with either NS or saline. Mean tumor sizes on treatment day (day 0) were, for the subcutaneous model, 140 ± 13.9 mm^3^ (mean ± standard error of the mean, SEM) for the surgery + NS group, 150 ± 9.9 mm^3^ for the surgery + saline group and 146.2 ± 12.8 mm^3^ for the surgery group. For the orthotopic model, 119.2 ± 10.8 mm^3^ for the surgery + NS group, 128.2 ± 11.2 mm^3^ for the surgery + saline group and 114.7 ± 5.8 mm^3^ for the surgery group. 

The animals were anesthetized, and a surgical scalpel (blade no. 15) was used to make an incision on the skin (and a second incision on muscle, if orthotopic) that allowed for tumor visualization. Next, ~75% of the tumor mass was weighed out and removed to mimic incomplete tumor resection. For this, it was assumed that 1 mm^3^ (as measured with MRI for the orthotopic model or CT for the subcutaneous model) corresponded to ~1 mg. Right after tumor removal, the mice were placed below an 807 nm diode laser beam (beam diameter of ~1 cm) and the surgical bed was exposed to NIR light for five minutes, at an intensity of 1.8 W/cm^2^ for subcutaneous tumors and 1.5 W/cm^2^ for orthotopic tumors. Maximum surface temperatures during irradiation were detected and recorded every 30 s with a thermal imaging camera (FLIRT420 Infrared Camera, FLIR systems, Täby, Sweden) and analyzed with FLIR Thermal Studio software. Afterwards, a 6.0 absorbable suture was used to close the incision. For pain relief, buprenorphine (0.3 mg/mL) was administered subcutaneously right before surgery and every six to eight hours for the next 24 h. Animals were monitored daily after the start of the treatment. For the mice bearing subcutaneous tumors, tumor size was measured every two or three days with the use of a caliper and the tumor volume was determined by the following formula: volume = ½ (length × width^2^). CT was only performed on treatment day for a more accurate determination of tumor size. When tumors reached ~1000 mm^3^, animals were euthanized. The study was terminated on day 40, when all remaining animals were euthanized. Mice bearing orthotopic tumors were MR-scanned every second day to follow tumor growth and euthanized when tumors reached ~800 mm^3^.

### 2.4. Tumor Size Monitoring with CT

Mice bearing subcutaneous tumors were anesthetized and placed inside a scanner (preclinical PET/CT Inveon, Siemens Medical Solutions, Malvern, PA, USA). CT images were acquired with the following parameters: 360 projections, 65 kV voltage, 500 μA current and 450 ms exposure. Regions of interest (ROIs) were manually drawn on the tumors using the Inveon Research Workplace software to determine tumor volume.

### 2.5. Tumor Size Monitoring with MRI

Mice bearing orthotopic tumors were anesthetized and placed inside a volume coil in a Bruker PharmaScan 7 T Small Animal MRI scanner. The TurboRare sequence was used, with the following parameters: repetition time = 2500 ms, echo time = 35 ms, field of view = 0.4 × 0.4 cm, image size = 256 × 256, slice thickness = 0.7 mm, 21 contiguous slices, and averages = 2. Both axial and coronal views were obtained from each tumor. Images were then analyzed with the free software HOROS; a ROI was drawn on every slice where the tumor was visible, and tumor volume was extracted.

### 2.6. Histology

HT1080 orthotopic tumors (~100 mm^3^; *n* = 3) were excised and fixed in paraformaldehyde 4% for later embedding in paraffin. Paraffin-embedded tissue blocks were sectioned in 4 μm slices with a microtome (Thermo Scientific Rotary Microtome Microm HM355S). The tissue slides were left to dry and then heated at 40 °C overnight and afterwards at 60 °C for one hour. Next, the slides were deparaffinized and rehydrated. For immunohistochemistry, the slides first underwent heat-induced antigen retrieval and afterwards they were positioned in coverplates and in Shandon racks (Thermo Fisher Scientific) for the actual staining procedure. Tissue sections were then blocked with peroxidase blocking solution (#S2023, Dako, Glostrup, Denmark) for 10 min, and with bovine serum albumin (BSA; 2% BSA in K-PBS, Sigma Aldrich, St. Louis, MO, USA) for 20 min. Primary antibodies, anti-Ki-67 (1:100; ab15580, Abcam, Cambridge, UK), anti-Vimentin (1:1000; HPA001762, Sigma Aldrich) and anti-CD-31 (1:250; ab28364, Abcam) were diluted in 2% BSA and incubated for 1 h. The slides were then incubated with an HRP-labelled polymer conjugated to secondary antibody (EnVision + System-HRP Labelled Polymer, Dako) anti-rabbit for 40 min. This was followed by DAB incubation (Liquid DAB + Substrate System™, Dako) for 5 min. Finally, the sections were counterstained with hematoxylin, dehydrated and mounted using an automated glass coverslipper (Dako).

Hematoxylin and eosin (HE) staining was performed after the deparaffinization and rehydration of tissue slides. Sections were stained with hematoxylin for 5 min and with eosin for 3 min. Sections were scanned on a Zeiss Axio Scan.Z1.

### 2.7. Biodistribution of Gold Nanoshells

Mice bearing subcutaneous (*n* = 5) or orthotopic (*n* = 4) HT1080 tumors were injected intravenously with gold NS (190 μL; 5.5 × 10^10^ NS/mL). The day after, mice were euthanized and tumor, spleen, liver, blood, kidney, and muscle were excised and stored at −20 °C until further use. For analysis of gold biodistribution, tissue (30–120 mg) was weighed out and digested in HNO_3_ (500 μL), HCl (50 μL) and H_2_O_2_ (300 μL) for at least six hours at 65 °C. Afterwards, 10 mL of MiliQ water was added and the tubes were weighed again to calculate the dilution factor. The samples were further diluted in 2% HCl containing 0.5 ppb ^193^Ir as the internal standard and subjected to inductively coupled plasma mass spectrometry or ICP-MS (ICAPq, Thermo Scientific, Hvidovre, Denmark) measurements. ^197^Au (0.125–1 ppb) was used, as standard. The instrument was operated as follows: RF power 1550 W and nebulizer gas flow 1.03 L min^−1^. For the intramuscular model, adjacent muscle refers to muscle located in the same leg as the tumor and distant muscle refers to muscle from the opposite leg.

### 2.8. Statistics and Data Analysis

Maximum temperatures at the last timepoint (300 s) for the surgery + NS and surgery + saline groups were compared using an unpaired *t*-test. The Kaplan–Meier method was used to create the survival curves. The percentage of tumor resected during surgery and the tumor volume on day 7 were compared using one-way ANOVA and a Tukey post hoc test. The data was plotted in Prism7 and shown as mean ± SEM.

## 3. Results

### 3.1. Subtotal Tumor Resection Followed by Photothermal Therapy in a Subcutaneous Fibrosarcoma Model

**Figure 1:**
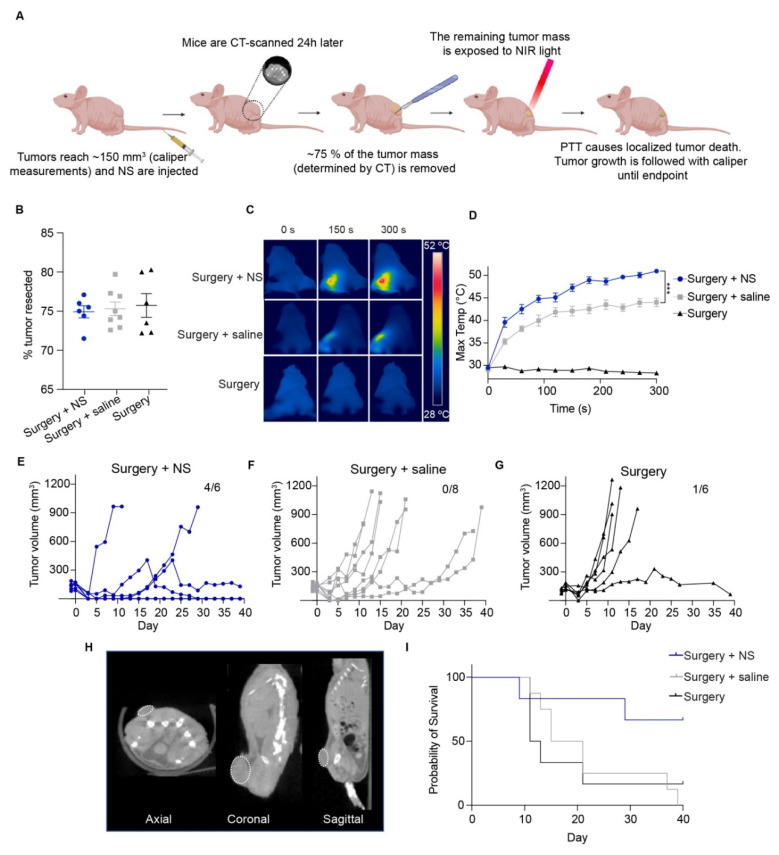
PTT as adjuvant to surgery in subcutaneous HT1080 tumors. (**A**) Mice were inoculated subcutaneously on the left flank with HT1080 cells. Tumors were measured with a caliper until they reached ~150 mm^3^, when the mice were divided into groups and injected with NS, saline or not injected (surgery group). The day after, mice were CT-scanned and ~75% of the tumor mass was resected. Immediately after, the surgical area was exposed to NIR light to perform PTT. The area was then closed, and tumor regrowth followed until endpoint (tumors reached ~1000 mm^3^ or study termination day). (**B**) Percentage of the tumor resected for each individual mouse from the different groups; surgery + NS (*n* = 6), surgery + saline (*n* = 8) and surgery (*n* = 6). Data shown as mean ± SEM. (**C**) Representative thermal images of maximum temperatures on the tumor surface throughout time. (**D**) Maximum temperatures for all groups. Surgery group represented by only 2 mice to show basal temperatures. *** represents *p* < 0.001. (**E**–**G**) Tumor growth curve for all groups. Study terminated on day 40, with four out of six surgery + NS mice still alive, none of the surgery + saline mice, and one of the mice in the surgery group. (**H**) Example of CT images taken right before surgery in axial, coronal and sagittal views. Tumors are marked with a dotted white line. (**I**) Survival curves for all groups.

First, we studied the feasibility of using PTT to ablate small tumor remnants left after surgery (Figure 1A). For this, mice bearing subcutaneous HT1080 tumors of ~150 mm^3^ were divided into three groups: mice injected with NS and undergoing combination treatment, i.e., surgery followed by PTT (surgery + NS group; *n* = 6); mice injected with saline and undergoing combination treatment (surgery + saline group; *n* = 8); and mice not injected with anything and undergoing surgery but no PTT (surgery group; *n* = 6).

First, mice were administered with NS or saline (day −1), and the day after injection (day 0) the mice were CT-scanned and underwent surgical removal of around 75% of the tumor mass (exact value obtained from the tumor measurement on the CT scan, Figure 1B). For the surgery + NS group, 74.9 ± 0.8% was removed, 75.4 ± 0.9% for the surgery + saline group, and 75.7 ± 1.5% for the surgery group. No significant differences in the percentage of tumor resected were observed between the groups. The residual tissue was subsequently exposed to NIR light intraoperatively to perform PTT, and the maximum temperatures on the tumor bed were recorded with a thermal imaging camera (Figure 1C,D).

We observed that the tumors from the surgery + NS group achieved temperature increases as high as what we have previously observed when PTT is performed as a standalone treatment [9]. The maximum temperatures after five minutes of irradiation were 50.7 ± 0.3 °C for the surgery + NS group and only 44 ± 0.9 °C for the surgery + saline group, probably due to unspecific heating caused by the laser. Temperatures in the surgery group correspond to basal temperatures since the tumors were not irradiated (Figure 1D). 

The higher temperatures reached by the surgery + NS group correlated with a delay in tumor growth (Figure 1E). The experiment was terminated on day 40. On this day, four out of six mice in the surgery + NS group were still alive, three of them with no visible tumor mass. No surgery + saline mice survived further this point, and only one of the surgery mice did (Figure 1F,G). In line with this, survival was significantly improved in the surgery + NS group (undefined median survival), while the surgery + saline and surgery groups experienced median survivals of 18 and 12 days respectively (Figure 1I). Figure 1H shows representative images from a CT scan for all planes.

### 3.2. Characterization of the Orthotopic Fibrosarcoma Model

**Figure 2:**
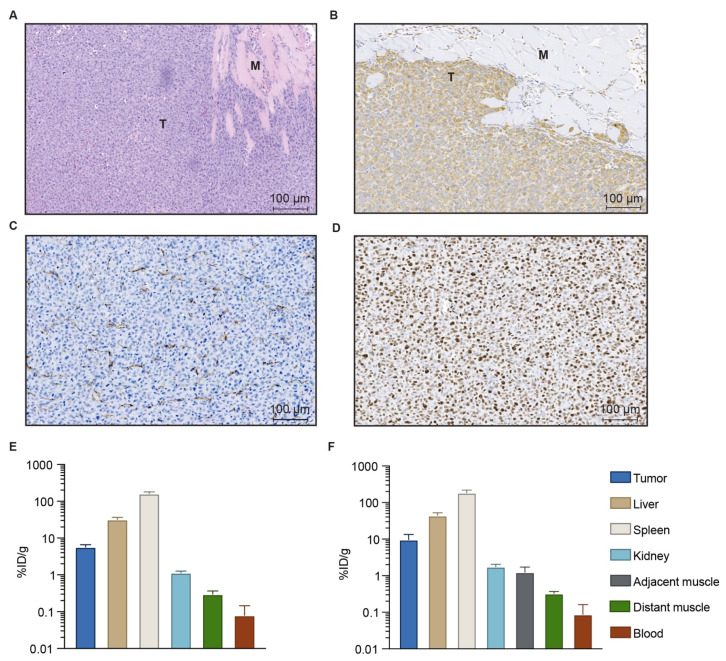
Histological properties of orthotopic HT1080 tumors and biodistribution of NS in tumor-bearing mice. (**A**) HE staining. T indicates tumor, M indicates muscle tissue. (**B**) Vimentin (fibrosarcoma marker) staining. (**C**) CD31 staining. (**D**) Ki-67 staining. Brown color denotes positive staining in (**B**–**D**). (**E**) NS biodistribution in mice bearing subcutaneous HT1080 tumors, measured using ICP-MS and expressed as percentage of injected dose per gram of tissue (%ID/g); *n* = 5. (**F**) NS biodistribution in mice bearing orthotopic HT1080 tumors, expressed as % ID/g; *n* = 4. Data shown as mean ± SEM.

Next, we evaluated the therapy in an orthotopic tumor model, where surgery better resembles the situation seen in the clinic. For this, HT1080 human fibrosarcoma cells were inoculated intramuscularly in the left thigh of the mice. When established this way, the HT1080 tumors were highly invasive. HE staining was performed and confirmed the cancer cells were infiltrating healthy muscle tissue, as observed on histology in the clinical setting. Figure 2A shows the HE staining of a ~100 mm^3^ HT1080 tumor, where the invasive tumor front presented finger-like structures penetrating muscle tissue. The tumors also presented a positive vimentin staining, characteristic of human fibrosarcoma and therefore useful to distinguish tumor from healthy tissue (Figure 2B). Additionally, CD31 (Figure 2C) and Ki-67 (Figure 2D) staining were performed to illustrate the highly angiogenic and fast-growing nature of this tumor model [22].

### 3.3. Biodistribution of Gold Nanoshells

In previous studies by us and others, the accumulation of NS was confirmed in different subcutaneous tumor models [9,23]. Here, we wanted to evaluate the presence of NS in the HT1080 orthotopic tumor model and compare it to the subcutaneous model. For this, NS were injected intravenously in mice bearing subcutaneous or intramuscular HT1080 tumors and left to distribute for 24 h to evaluate NS biodistribution at the time established for PTT. The day after, the mice were euthanized and tumor, spleen, liver, blood, kidney and muscle were obtained. The amount of Au was then measured using ICP-MS (Figure 2E,F) to determine the percentage of injected dose per gram of tissue (% ID/g). For the intramuscular model, both adjacent muscle tissue (refers to the muscle tissue where the tumor was inoculated) and distant muscle tissue (refers to muscle from the opposite leg) were excised. The muscle obtained from the subcutaneous tumor-bearing mice is noted as distant muscle in Figure 2E for simplification.

This analysis confirmed the accumulation of NS in both subcutaneous (Figure 2E) and intramuscular (Figure 2F) models. Interestingly, there was a slight tendency for the NS to accumulate more in the orthotopic intramuscular tumors (9.67 ± 3.8% ID/g) when compared to subcutaneous tumors (5.73 ± 0.9% ID/g). This corresponded to 18.14 ± 7 µg of Au per gram of wet tissue for the orthotopic tumors and 10.7 ± 1.7 µg of Au/g wet tissue for the subcutaneous tumors. As expected for the other organs, NS ended up primarily in spleen, followed by liver and in a much lower concentration, kidney. Very low amounts of Au were detected in the blood and muscle 24 h after injection. For the orthotopic model in particular, we observed a higher accumulation in the muscle where the tumor was located (i.e., adjacent muscle, 1.24 ± 0.5% ID/g) compared to the muscle present in the opposite leg (distant muscle, 0.52 ± 0.05% ID/g).

### 3.4. Subtotal Tumor Resection Followed by Photothermal Therapy in an Orthotopic Fibrosarcoma Model

**Figure 3:**
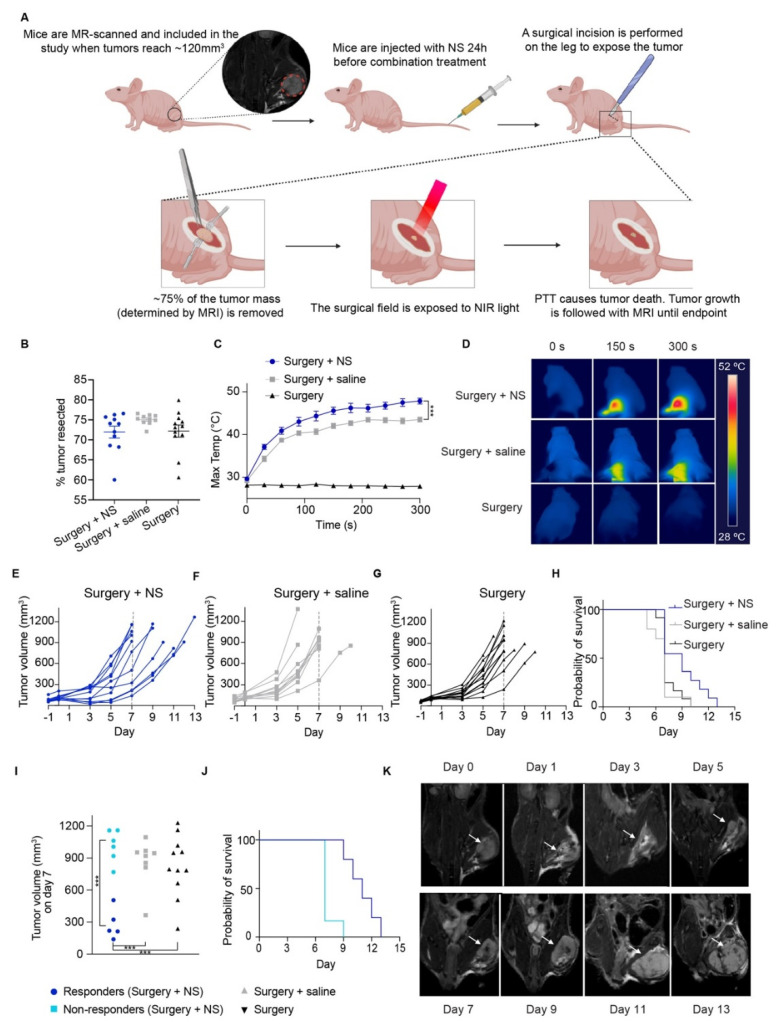
PTT as adjuvant to surgery in orthotopic HT1080 tumors. (**A**) Mice were inoculated with HT1080 cells intramuscularly, in the left upper leg. Tumor growth was followed with MRI, and the mice were included in the study when tumors reached ~120 mm^3^. Mice were then injected with NS, saline, or not injected (surgery group). The following day, ~70–75% of the tumor mass was removed, and immediately after the surgical area was exposed to NIR laser light. The incision was closed, and the mice were scanned every other day until tumors reached over 800 mm^3^, when they were euthanized**.** (**B**) Percentage of the tumor taken out during surgery, based on its measurement on MRI. Data shown as mean ± SEM. (**C**) Maximum temperatures on the tumor surface during PTT for all groups. Surgery group represented only by 3 mice to show basal temperatures. *** represents *p* < 0.001. (**D**) Representative thermal images of maximum temperatures on the tumor surface over time. (**E**–**G**) Tumor growth curve for the surgery + NS (*n* = 11), surgery + saline (*n* = 10) and surgery (*n* = 12) groups respectively. (**H**) Survival curves for all groups. (**I**) Tumor volume on day 7 (three mice not included as they were euthanized before day 7), with the surgery + NS group divided into responders (dark blue) and non-responders (light blue) to treatment. (**J**) Survival curves for responders and non-responders. (**K**) Representative MRI images throughout time in a surgery + NS mouse. White arrows point to the tumor.

After inoculation, mice bearing orthotopic tumors were MR-scanned to follow tumor growth, and animals were included in the study when tumors reached ~120 mm^3^. Then, mice were divided into three groups as for the subcutaneous model; surgery + NS group (mice receiving NS and combination treatment, i.e., surgery and PTT; *n* = 11), surgery + saline group (mice receiving saline and the combination treatment; *n* = 10), and surgery group (mice not injected and undergoing surgery but no PTT; *n* = 12). The study design is depicted in Figure 3A.

One day before treatment (day −1), mice were injected with NS (surgery + NS group), saline (surgery + saline group) or not injected (surgery group). The day after (day 0), mice had an MR scan followed by surgery. Around 70–75% of the entire tumor mass was weighed out and removed (72 ± 1.5% for the surgery + NS group, 75.1 ± 0.4% for the surgery + saline group and 72.2 ± 1.5% for the surgery group; Figure 3B), leaving only a small remnant to mimic incomplete tumor removal during surgery in a clinical setting. No significant differences in the percentage of tumor resected were observed between the groups. Right after, the surgical field was exposed to NIR light for the surgery + NS and surgery + saline groups (Figure 3C,D). As we had observed in the subcutaneous model, the maximum surface temperatures reached on the orthotopic tumors bearing NS were significantly higher than the temperatures achieved in the surgery + saline group, being 47.8 ± 0.7 °C for the surgery + NS group and 43.5 ± 0.7 °C for the surgery + saline group. The mice in the surgery group were also placed under the NIR laser (turned off) after surgery to mimic the process.

The higher temperatures recorded for the surgery + NS group correlated with a tendency towards slower tumor growth and elongated survival when compared to surgery + saline and surgery groups (Figure 3E–H). The median survival was 9 days for the surgery + NS group and 7 days for both surgery + saline and surgery groups. Even though the treatment effect was apparent, it was not as pronounced as for the subcutaneous model, probably also because the orthotopic tumors duplicated their size at a very fast rate, making it difficult to detect the treatment effect long-term. Nevertheless, we could clearly see two separated clusters in the surgery + NS group, one that responded to the treatment and presented elongated survival, and one where the treatment effect matched the effect found in the two control groups. These two groups could be distinguished by their tumor volumes (according to the median) measured on the MRI scans on day 7; the responders to treatment presented a significantly smaller tumor size when compared to the cluster of non-responder surgery + NS mice, and to the mice in both the surgery + saline and surgery groups (Figure 3I). This could also be observed in the survival curves of the two subgroups, where the responders presented a median survival of 11 days, compared to 7 days for the non-responders (Figure 3J). Finally, Figure 3K depicts representative MR images at different time points for a mouse in the surgery + NS group to illustrate tumor growth.

## 4. Discussion

Despite the continuous development of novel anticancer strategies, surgery has remained the most important treatment modality for solid tumors, and refinements of surgical procedures have improved outcomes for many types of cancer. In recent years, much effort has been put into developing strategies that could improve the outcome of surgery when complete resection is not technically possible (e.g., central nervous system tumors) or when surgical removal fails to completely eradicate the tumor [24].

PTT has previously shown promising results as an adjuvant treatment in combination with radiation and chemotherapy [25]. However, PTT also has potential as an adjuvant therapy during or after tumor resection surgery. Importantly, Wang et al. [13] recently applied PTT as an alternative to radiation therapy in combination with breast-conserving surgery in an orthotopic breast tumor model. This new approach could be particularly relevant in patients that are susceptible to radiation damage.

Fibrosarcoma is a rare and very aggressive tumor mainly treated with surgery due to its resistance to other conventional therapies. However, tumor recurrence, facilitated by the infiltrative character of the tumor that complicates getting clear margins, is still a major challenge. Thus, in this study we investigated the possible impact of PTT as adjuvant to surgery on outcomes in a highly invasive human fibrosarcoma (HT1080) mouse model. First, to evaluate the feasibility of the approach, we performed subtotal tumor removal (R2 = macroscopically involved tumor margin [26]) in subcutaneous tumors to mimic a clinical setting where it is not possible to achieve complete removal of the tumor during surgery. Next, we performed NS-based PTT to investigate whether it was possible to generate hyperthermia-induced damage on the tumor remnants. We observed that the temperatures achieved during PTT on residual cancerous tissue were high enough to cause irreversible tissue damage (i.e., over 48 °C [6]) in the group bearing NS, but not in the mice injected with saline. This confirmed that the remaining NS were still able to generate heat for successful PTT in the small residual tumor deposits, with a subsequent slowdown in tumor growth and even complete tumor disappearance for some animals. It should be noted that deliberately leaving macroscopic tumor residue behind is an artificial way of mimicking incomplete clinical tumor resections, which in human patients consist of microscopic tumor deposits in the surgical bed. However, we find this approach was suitable as a first step to evaluate the feasibility of applying PTT as part of the procedure where the tumor is resected, as well as for determining whether this type of combination treatment could potentially be valuable. Moreover, this approach has previously been used in similar studies [1,14,27]. For instance, the use of the fluorescent photosensitizer IRDye700DX conjugated to panitumumab (anti-EGFR antibody) to perform photoimmunotherapy after incomplete surgical resection of subcutaneous tumors has been studied [1].

After confirming that the NS still triggered high temperatures even in small tumor remnants in the subcutaneous tumor model, we proceeded to test this strategy in fibrosarcoma tumors established in the muscular tissue of the left thigh of mice, an orthotopic model that has previously been shown to mimic the highly aggressive and infiltrative tumor progression seen in patients [22]. This was confirmed by the histological assessment of the orthotopic tumor. In addition, the presence of NS in the orthotopic tumors turned out to be slightly higher than in subcutaneous tumors, probably due to the increased vascular perfusion that characterizes orthotopic models [28]. We also observed a higher presence of NS in the muscle tissue where the tumor was embedded compared to control muscle. This was also expected due to the infiltrative character of the tumor.

For the orthotopic model, we used MRI to follow tumor growth, which also proved valuable to observe treatment effect over time. Of the animals undergoing surgery and NS-based PTT, we found a cluster of mice that responded to the treatment and presented slower tumor growth and elongated survival. However, there was also a cluster of non-responding mice with no benefit from the therapy and tumor growth comparable to the control groups. There can be different explanations for the heterogenous response. For instance, the HT1080 fibrosarcoma tumor model chosen for this study grows at an extremely high rate when established orthotopically (tumor size can double every two days), which can result in heterogenous treatment outcomes. Also, the location of the fibrosarcoma mass (embedded in muscle tissue) hinders light penetration from the surgical incision into the deeper parts of the tumor remnants. The infiltrative growth and high vascularity of the HT1080 orthotopic tumors, as shown in our histological analysis and in the literature [22], resulted in frequent bleeding in the tumor bed during surgery. This caused an inaccurate reading of maximum temperatures by the thermographic camera and possibly influenced the irradiation of the remaining tumor tissue, since hemoglobin is also efficient at absorbing light. In contrast, the orthotopic breast tumor models used in the literature, where PTT has previously shown promising results, were located on the surface of the animal, a situation comparable to the subcutaneous tumor models [13,15].

Additionally, there are multiple technical parameters involved in PTT, such as the diameter of the area covered by the laser, nanoparticle delivery and heat distribution, and they all can influence treatment outcome. The NS used here are particularly interesting for clinical translation since they are already being used in clinical settings [29]. However, the NS accumulate in the tumor only passively through the enhanced permeability and retention (EPR) effect, due to special tumor properties such as leaky vasculature and impaired lymphatic drainage [30]. The uptake could be enhanced using a tumor-targeted photothermal agent to achieve more tumor-specific delivery of energy to the tumor compartment [31]. Also, the treatment effect could potentially be improved in some of the non-responding animals by increasing the laser intensity, as the powers we used here were slightly lower than in the subcutaneous study (1.5 W/cm^2^ vs. 1.8 W/cm^2^) due to the higher degree of invasiveness of the surgical procedure. However, increased delivery of energy to the tumor compartment may also increase the risk of damage to adjacent healthy tissues. 

We believe the results presented here show the potential of using PTT as an adjuvant to surgery in a xenograft mouse tumor model that closely mimics the aggressive tumor progression seen in the clinic. Moreover, this tumor is located in areas of difficult access and where laser light exposure is only possible during surgery, making it a two-in-one treatment. In a clinical setting, the results showed here would translate into an additional intraoperative treatment of microscopic tumor deposits left in the tumor bed after primary resection. As a future perspective, a tumor-targeted fluorescence-based surgical approach combined with PTT could further improve treatment effects. The fluorescent probe could visually guide tumor resection by lighting up all the cancerous tissue and enable the detection of possible residual disease in the surgical field. Thereafter, fluorescent tumor residues could undergo PTT due to the light-absorbing/heat-generating properties of the fluorescent probe. Studies have shown the potential of applying clinically approved fluorophore ICG for this purpose [32,33,34].

## 5. Conclusions

In this study, tumor surgery followed by PTT improved treatment outcome in a mouse model of fibrosarcoma. Thus, the combination of PTT and surgery represents a promising approach for aggressive tumors and in cases where complete surgical resection is not achievable. Clinical translation of this method should come hand-in-hand with optimization of the PTT conditions, and possibly taking advantage of the benefits provided by optical image-guided surgery.

## Data Availability

The raw data are available upon request.
